# Adoptive Transfer of Lepr^+^ Bone Marrow Cells Attenuates the Osteopetrotic Phenotype of *db*/*db* Mice

**DOI:** 10.3390/ijms26115120

**Published:** 2025-05-27

**Authors:** Russell T. Turner, Carmen P. Wong, Kenneth A. Philbrick, Jessica A. Keune, Edwin M. Labut, Scott A. Menn, Adam J. Branscum, Urszula T. Iwaniec

**Affiliations:** 1 Skeletal Biology Laboratory, School of Nutrition and Public Health, Oregon State University, Corvallis, OR 97331, USAcarmen.wong@oregonstate.edu (C.P.W.); kenneth.philbrick@gmail.com (K.A.P.); ed.labut@oregonstate.edu (E.M.L.); 2 Center for Healthy Aging Research, Oregon State University, Corvallis, OR 97331, USA; 3 Radiation Center, Oregon State University, Corvallis, OR 97331, USA; scott.menn@oregonstate.edu; 4 Biostatistics Program, School of Nutrition and Public Health, Oregon State University, Corvallis, OR 97331, USA; adam.branscum@oregonstate.edu

**Keywords:** leptin, cartilage, osteopetrosis, skeletal maturation

## Abstract

Leptin-deficient (*ob*/*ob*) and leptin receptor (Lepr)-deficient *db*/*db* mice develop a mild form of osteoclast-rich osteopetrosis, most evident in long bone epiphyses, implying leptin is important for normal replacement of cartilage during skeletal maturation. However, it is unclear whether leptin acts as a permissive or regulatory factor and whether its actions are mediated via peripheral pathways. Here we show the osteopetrotic phenotype is not evident in *ob*/+ or *db*/+ mice, suggesting that leptin acts as a critical but permissive factor for skeletal maturation. The importance of leptin is further supported by our results showing that interventions known to increase bone resorption (mild cold stress, simulated microgravity, or particle-induced inflammation) did not advance skeletal maturation in *ob*/*ob* mice whereas long-duration hypothalamic leptin gene therapy was effective. Additionally, administration of leptin by subcutaneously implanted osmotic pumps (400 ng/h) for 2 weeks accelerated skeletal maturation in *ob*/*ob* mice. Because leptin has the potential to act on the skeleton through peripheral pathways, we interrogated osteoclast-lineage cells for the presence of Lepr and evaluated skeletal response to the introduction of bone marrow Lepr^+^ cells into *db*/*db* mice. We identified Lepr on marrow MCSFR^+^CD11b^+^ osteoclast precursors and on osteoclasts generated in vitro. We then adoptively transferred Lepr^+^ marrow cells from GFP mice or wildtype (WT) mice into Lepr^-^ *db*/*db* mice. Following engraftment, most MCSFR^+^ CD11b^+^ cells in marrow expressed GFP. Whereas *db*/*db*→*db*/*db* had minimal influence on epiphyseal cartilage, WT→*db*/*db* decreased cartilage. These findings suggest peripheral leptin signaling is required for normal osteoclast-dependent replacement of cartilage by bone during skeletal maturation.

## 1. Introduction

Studies investigating leptin-deficient *ob*/*ob* and leptin receptor (Lepr)-deficient *db*/*db* mice have shown that functional leptin signaling is important for normal linear bone growth, bone mass accrual, and bone turnover balance. Bone mass is generally positively associated with body weight [1]. However, despite morbid obesity, *ob*/*ob* mice have reduced whole body bone mineral content [2,3,4,5,6], reduced linear growth [7], reduced mechanical strength of the femur [2,5,8,9], and reduced biochemical and histomorphometric indices of bone turnover [10,11]. Reduced epiphyseal growth in long bones of leptin signaling-deficient mice is associated with severe abnormalities in growth plate architecture and mild osteopetrosis [11,12,13,14].

While the importance of leptin signaling in bone physiology is well recognized, it has proven difficult to dissociate direct effects of the adipokine on target cells in bone from indirect effects mediated via the central nervous system. This is due, in part, to ambiguities inherent to the simultaneous transport of leptin across the blood–brain barrier into the brain and movement of leptin from the brain into peripheral circulation. As described by Banks et al. [15], leptin is translocated from peripheral circulation across the blood–brain barrier into the hypothalamus by a saturable transport process. Less appreciated is the fact that leptin delivered into the hypothalamus returns to peripheral circulation via a non-saturable pathway (spinal fluid resorption) at levels capable of impacting leptin target cells [15,16,17]. These observations indicate that leptin, whether delivered into the peripheral circulation or centrally, is capable of influencing bone metabolism by multiple routes of action.

Additionally, white adipose tissue (WAT), brown adipose tissue (BAT), the hypothalamus, skeletal muscle, reproductive tissues, the pancreas, adrenal glands, and the immune system respond to leptin [18]. Changes in these extra-skeletal leptin target organs in the leptin signaling-deficient state can influence bone metabolism due to hypogonadism, elevated corticosteroid levels, type 2 diabetes, sarcopenia, impaired thermoregulation, and/or greatly increased body weight [19,20,21]. To minimize some of these confounders, we performed studies at a thermoneutral temperature (30–32 °C) to control for impaired thermoregulation and pair-fed *ob*/*ob* mice to WT mice to control for skeletal effects of excess weight and hyperglycemia [20]. These studies showed that disturbances in energy balance and thermoregulation contribute to the skeletal phenotype of leptin signaling-deficient mice [20]. Thus, to establish the specific actions on bone, it is necessary to dissociate peripheral from central pathways and minimize, if not eliminate, confounding actions of the adipokine on other target organs.

We used two complementary approaches to identify the relevant pathway(s) for leptin action on bone: (1) differential dose–response effects of leptin on bone and energy metabolism in *ob*/*ob* mice [11] and (2) adoptive transfer of Lepr^-^ cells obtained from the bone marrow of leptin receptor-deficient *db*/*db* mice into WT mice [10]. The former method is based on the premise that peripheral actions of leptin occur at lower dose rates than central actions because of the limited transport of leptin from peripheral circulation across the blood–brain barrier into the brain. The latter method selectively introduces Lepr^-^ cells from the bone marrow of *db*/*db* mice into the bone microenvironment of WT mice [10]. Both methods yielded results strongly supporting the concept that leptin positively regulates osteoblast differentiation and activity at low circulating levels that have minimal effects on the central nervous system. However, neither of the studies addressed the effects of leptin on osteoclast function.

Osteoclasts located adjacent to mineralized cartilage (often referred to as chondroclasts) originate from hematopoietic stem cells (HSCs) and play a key role in bone accrual during endochondral ossification [22]. Leptin signaling deficiency results in a mild form of osteoclast-rich osteopetrosis that persists long after normal skeletal maturation [12]. However, it is not clear whether leptin acts as a permissive factor and, if so, whether generalized or focal increases in bone resorption are sufficient to correct the deficit in skeletal maturation in leptin signaling-deficient mice. It is also not clear whether leptin acts on chondroclasts via peripheral or central regulatory pathways to increase resorption of the cartilage template to advance skeletal maturation. To address these questions, we investigated the impact of partial leptin signaling deficiency using *ob*/+ and *db*/+ mice and several methods for exaggerating bone resorption (cold temperature stress, simulated microgravity, polyethylene particle-induced inflammation) on skeletal maturation in *ob*/*ob* mice. In addition, we determined whether osteoclast-lineage cells express Lepr and whether adoptive transfer of Lepr^+^ bone marrow cells into Lepr^-^ *db*/*db* mice corrects pathological retention of calcified cartilage.

## 2. Results

### 2.1. Histopathology Evaluation of Distal Femur Epiphysis in 4-Month-Old WT, ob/ob, ob/+, db/db, and db/+ Mice

The goal of these studies was to confirm delayed skeletal maturation in the distal femur epiphysis in female *ob*/*ob* mice and to determine if skeletal maturation is delayed in male *ob*/*ob* mice and in partial leptin signaling-deficient male *ob*/+ and female *db*/+ mice.

**Figure 1:**
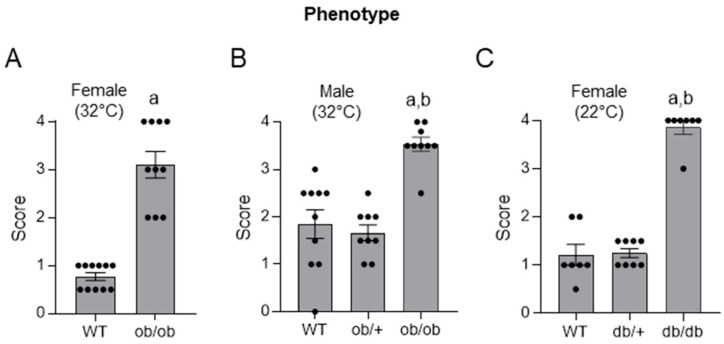
Skeletal maturation in distal femur epiphysis in female WT and *ob*/*ob* mice housed at ther-moneutral temperature (**A**); in male WT, ob/+, and *ob*/*ob* mice housed at thermoneutral temperature (**B**); and in female WT, *db*/+, and *db*/*db* mice housed at room temperature (**C**). Data are mean ± SE with dots representing individual data points. *N* = 10–11/group (**A**), 9–10/group (**B**), and 7–8/group (**C**). ^a^ Different from WT, *p* ≤ 0.05; ^b^ Different from *ob*/+ (**B**) or *db*/+ (**C**), respectively, *p* ≤ 0.05. Data were analyzed using *t*-test (**A**) or one-way ANOVA (**B**,**C**).

As shown in **Panel A** of **Figure 1**, the histopathology score in the distal femur epiphysis was higher in female *ob*/*ob* mice compared to female WT mice. This finding confirms published results using an alternative evaluation of epiphyseal calcified cartilage [20]: the correlation coefficient for measurements (*n* = 31) performed using the two methods was 0.86. As shown in **Panel B**, the histopathology score was higher in male *ob*/*ob* mice compared to male WT mice or male *ob*/+ mice. Significant differences in score were not detected between the *ob*/+ and WT mice. As shown in **Panel C**, the histopathology score was higher in female *db*/*db* mice than in female WT or *db*/+ mice. Significant differences in score were not detected between the *db*/+ and WT mice.

### 2.2. Impact of Interventions Known to Increase Bone Resorption on Histopathology Score in ob/ob Mice

Delayed skeletal maturation in *ob*/*ob* and *db*/*db* mice is due to decreased osteoclast activity [10,20]. Therefore, we investigated the effects of increased bone resorption induced by cold stress (room temperature housing) [23], simulated microgravity using HU [24], and polyethylene particle-induced focal inflammation [25] on skeletal maturation in the distal femur epiphysis. In addition, we evaluated the effects of polyethylene particle-induced focal inflammation, with or without leptin.

**Figure 2:**
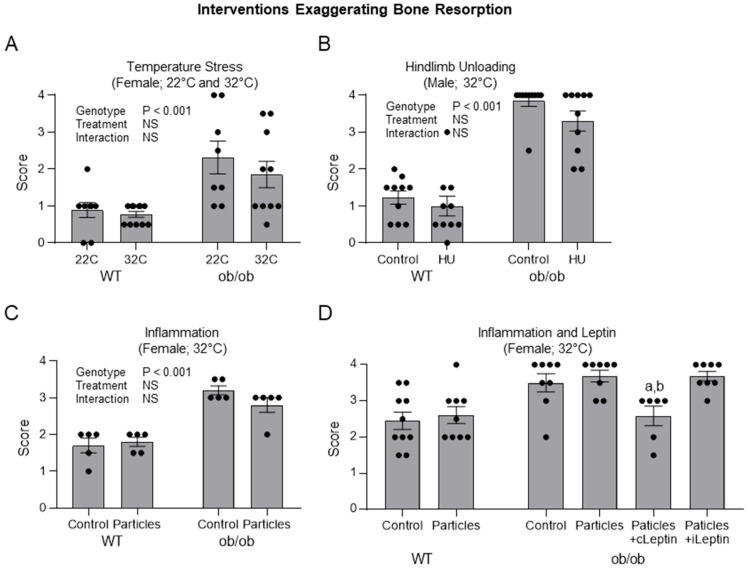
Skeletal maturation in distal femur epiphysis in WT and *ob*/*ob* mice subjected to interventions that exaggerate bone resorption, including room temperature housing (**A**), hindlimb unloading (**B**), and particle-induced inflammation (**C**), and in *ob*/*ob* mice subjected to particle-induced inflammation and administered either continuous leptin (cLeptin) or intermittent leptin (iLeptin) (**D**). Data are mean ± SE with dots representing individual data points. *N* = 8–11/group (**A**), 10/group (**B**), 5/group (**C**), and 6–10/group (**D**). NS, not significant. Panel D, ^a^ Different from *ob*/*ob* + Particles, *p* ≤ 0.05; ^b^ Different from *ob*/*ob* + Particles + iLeptin, *p* ≤ 0.05. Data were analyzed using two-way ANOVA (**A**–**C**) or one-way ANOVA (**D**).

As shown in **Panel A** of **Figure 2**, the histopathology score was higher in female *ob*/*ob* mice compared to female WT mice, irrespective of housing temperature. As shown in **Panel B**, the histopathology score was higher in male *ob*/*ob* mice compared to male WT mice, irrespective of HU. As shown in **Panel C**, the histopathology score was higher in female *ob*/*ob* mice compared to female WT mice, irrespective of particle-induced inflammation. However, as shown in **Panel D**, treatment with particles plus continuous, but not intermittent, leptin resulted in a lower histopathology score in the female *ob*/*ob* mice. This latter finding is consistent with the reduced efficacy of intermittent leptin on bone.

### 2.3. Effects of Leptin Replacement on Histopathology Score in ob/ob Mice

**Figure 3:**
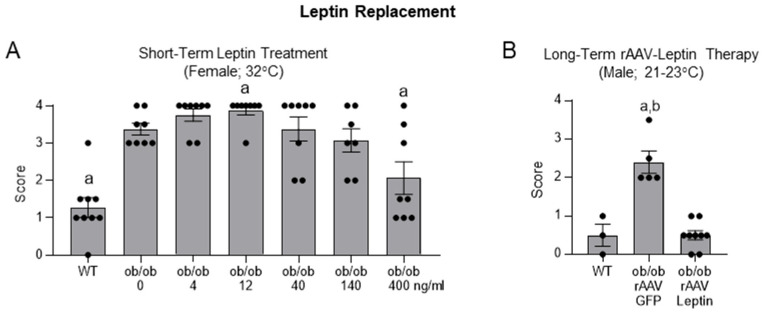
Skeletal maturation in distal femur epiphysis in female *ob*/*ob* mice housed at thermoneutral temperature in response to short-term (2 weeks) administration of subcutaneous leptin (**A**) and in male *ob*/*ob* mice housed at room temperature in response to long-term (15 weeks) hypothalamic leptin gene therapy (rAAV-Leptin) (**B**). Data are mean ± SE with dots representing individual data points. *N* = 7–8/group (**A**) and 3–9/group (**B**). Panel A: ^a^ Different from *ob*/*ob* + 0 ng/hr leptin, *p* ≤ 0.05. Panel B, ^a^ Different from WT, *p* ≤ 0.05; ^b^ Different from *ob*/*ob* + rAAV Leptin, *p* ≤ 0.05. Data were analyzed using one-way ANOVA.

Continuous subcutaneous delivery of leptin to *ob*/*ob* mice normalizes appetite within 3 days and induces weight loss within 7 days [25]. We therefore determined if leptin administration for 2 weeks accelerates skeletal maturation. The dose–response effects of subcutaneous infusion of leptin on the histopathology score in female *ob*/*ob* mice are shown in **Panel A** of **Figure 3**. The histopathology score was lower in WT mice and *ob*/*ob* mice treated with leptin at a dose rate of 400 ng/h compared to vehicle-treated *ob*/*ob* mice. The score was higher in mice treated with leptin at a dose rate of 12 ng/h compared to the vehicle-treated mice. The presence of calcified cartilage in the epiphysis of *ob*/*ob* and *db*/*db* mice represents a delayed skeletal maturation, not the generation of new chondrocytes. The slightly higher values in the 12 ng/d *ob*/*ob* mice compared to vehicle-treated *ob*/*ob* mice implies that less cartilage was resorbed during the 2-week experiment. Significant differences in the histopathology score were not detected between vehicle-treated *ob*/*ob* mice and *ob*/*ob* mice treated at leptin dose rates of 4, 40, or 140 ng/h.

Long-duration (30 weeks) hypothalamic leptin gene therapy advanced skeletal maturation in male *ob*/*ob* mice [12]. This finding suggests that normalizing leptin signaling in these mice will ultimately correct the osteopetrotic phenotype. Here we obtained additional support for this conclusion. The effect of 15 weeks of hypothalamic leptin gene therapy on skeletal maturation in male *ob*/*ob* mice is shown in **Panel B**. The histopathology score was higher in rAAV-GFP *ob*/*ob* mice than rAAV-Leptin *ob*/*ob* mice or WT mice. Significant differences in score were not detected between WT mice and rAAV-Leptin *ob*/*ob* mice, indicating that 15 weeks of transgene expression fully reversed the osteopetrotic skeletal phenotype in these animals.

### 2.4. Expression of Lepr by Hematopoietic Lineage Cells

**Figure 4:**
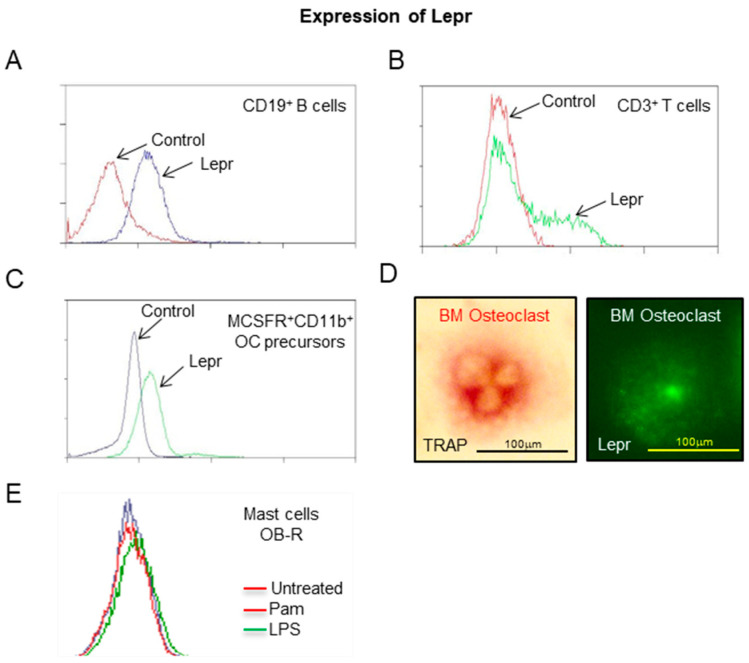
Expression of Lepr by hematopoietic lineage cells in WT (B6) mice. Lepr was detected in splenic CD19^+^ B cells (**A**) and in CD3^+^ T cells following 48h in vitro activation with anti-CD3 (**B**). MCSFR^+^ CD11b^+^ cells (osteoclast precursors) from bone marrow were positive for Lepr expression (**C**). Additionally, osteoclasts differentiated in vitro from bone marrow were positive for Lepr (**D**). Mast cells differentiated in vitro from bone marrow did not express Lepr with or without 24h Pam3CSK1 (Pam; TLR2 agonist) or LPS (TLR4 agonist) stimulation (**E**).

As shown in **Figure 4**, Lepr was detected in splenic CD19^+^ B cells (**Panel A**) and in CD3^+^ T cells 48h following activation with anti-CD3^+^ (**Panel B**). Osteoclast precursor cells (MCSFR^+^ CD11b^+^) from the bone marrow of WT mice were positive for Lepr (**Panel C**). Additionally, bone marrow-derived osteoclasts generated in vitro were positive for Lepr (**Panel D**). Bone marrow-derived mast cells differentiated in vitro did not express Lepr with or without Pam3CSK1 (TLR2) or LPS (TLR4) agonist stimulation (**Panel E**).

### 2.5. Efficacy of Adoptive Transfer of GFP-Expressing Bone Marrow Cells into WT and db/db Mice

**Figure 5:**
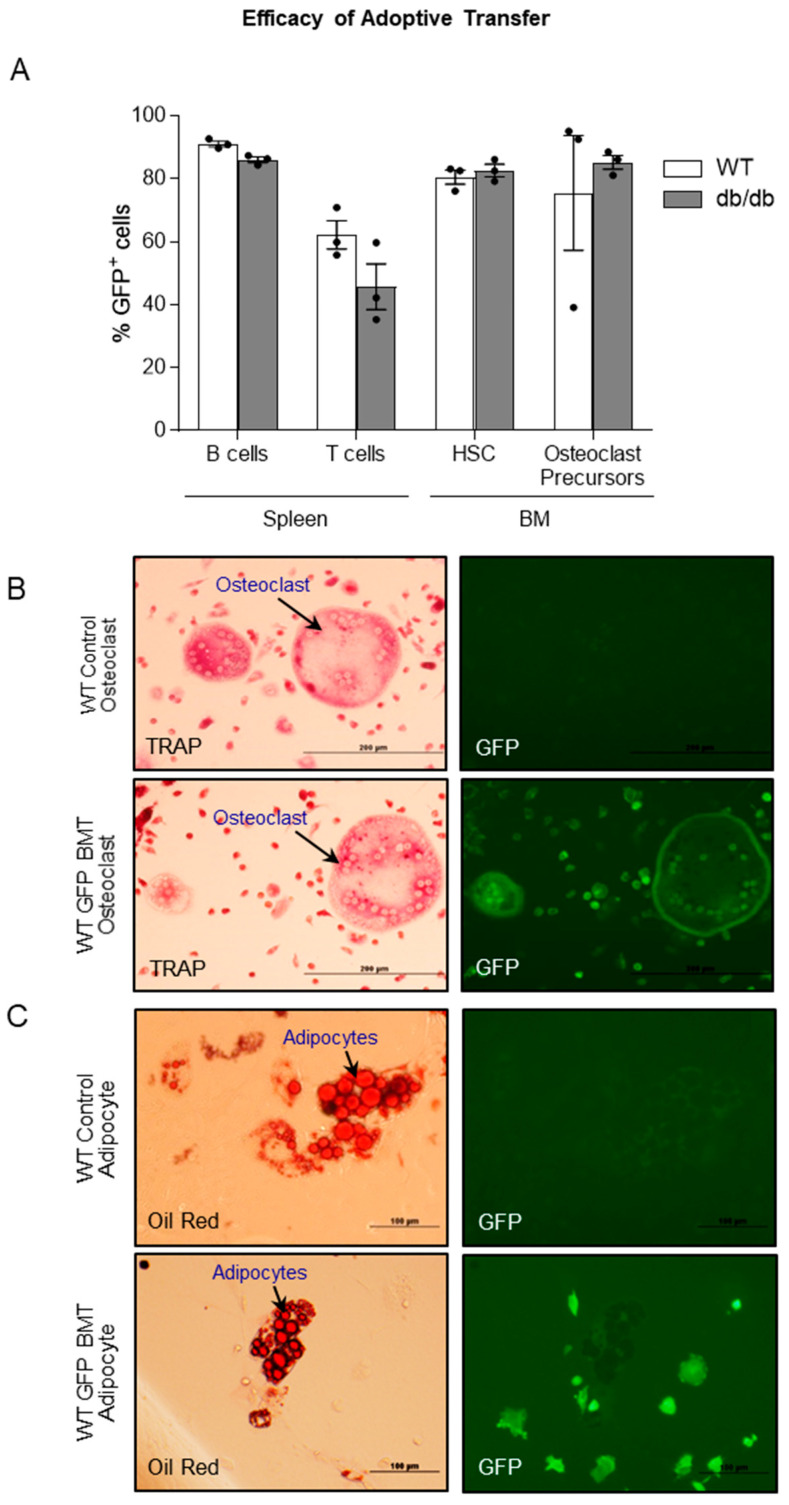
Efficacy of adoptive transfer of GFP bone marrow cells into irradiated WT and *db*/*db* mice. Efficient reconstitution of B cell (CD19^+^) and T cell (CD3^+^) populations in spleen and HSCs (Lineage^-^Sca1^+^cKit^+^) and osteoclast precursor cells (MCSFR^+^ CD11b^+^) in bone marrow was observed in WT as well as *db*/*db* mice (**A**). Additionally, osteoclasts differentiated in vitro from bone marrow of WT mice following GFP bone marrow adoptive transfer expressed GFP (**B**). In contrast, adipocytes generated from bone marrow of WT mice following adoptive transfer of GFP bone marrow did not express GFP (**C**). Note adjacent cells in the same field that were negative for Oil Red^+^ lipid droplets were GFP^+^. Data are mean ± SE with dots representing individual data points. *N* = 3/group (**A**).

The efficacy of the reconstitution of the immune system in lethally irradiated mice was evaluated following adoptive transfer of bone marrow from B6.GFP mice into WT and *db*/*db* mice. As shown in **Panel A** of **Figure 5**, efficient reconstitution of B and T cell populations in spleen as well as HSC and osteoclast precursor cells in bone marrow was observed in *db*/*db* as well as WT mice. Additionally (**Panel B**), most osteoclasts generated from the bone marrow of WT mice following adoptive transfer of GFP bone marrow expressed GFP. In contrast (**Panel C**), few adipocytes generated from the bone marrow of WT mice following adoptive transfer of GFP bone marrow expressed GFP. The latter finding suggests adoptive transfer of bone marrow cells into lethally irradiated recipients primarily reconstituted the hematopoietic compartment (osteoclasts) and not the mesenchymal stromal cell compartment (adipocytes) in recipient mice.

### 2.6. Effects of Adoptive Transfer of WT and db/db Bone Marrow Cells on Histopathology Score in WT and db/db Mice

**Figure 6:**
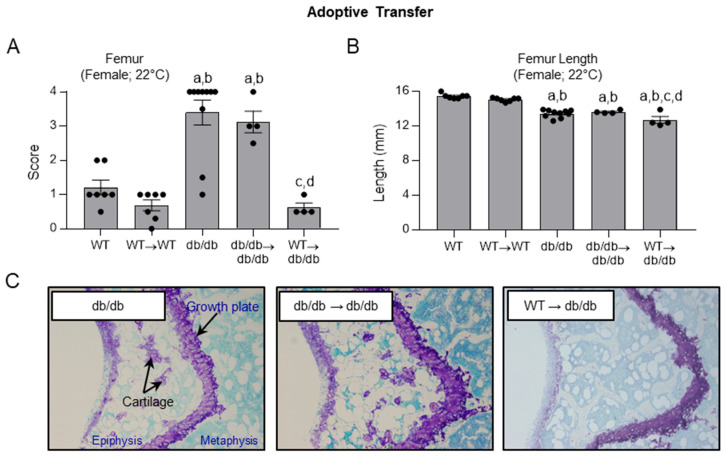
Effect of adoptive transfer of female WT (Lepr^+^) bone marrow cells into female *db*/*db* (Lepr) mice (WT→*db*/*db*) on skeletal maturation in distal femur epiphysis (**A**) and on femur length (**B**). Control mice received bone marrow cells from the same genotype (WT→WT, and *db*/*db*→*db*/*db*, respectively). Representative micrographs of cartilage in distal femur epiphysis in *db*/*db*, *db*/*db*→*db*/*db*, and WT→*db*/*db* mice are shown in panel (**C**). Data are mean ± SE. N = 4–10 /group with dots representing individual data points. ^a^ Different from WT, *p* ≤ 0.05; ^b^ Different from WT → WT, *p* ≤ 0.05; ^c^ Different from *db*/*db*, *p* ≤ 0.05; ^d^ Different from *db*/*db* → *db*/*db*, *p* ≤ 0.05. Data were analyzed using one-way ANOVA.

The goal of this study was to determine the impact of adoptive transfer of Lepr^+^ (WT) and Lepr^-^ (*db*/*db*) bone marrow cells on skeletal maturation in recipient mice. As shown in **Panel A** of **Figure 6**, untreated female *db*/*db* mice had a higher histopathology score in the distal femur epiphysis than untreated WT mice, and this relationship was not altered 8 weeks following adoptive transfer of *db*/*db*→*db*/*db* or WT→WT. In contrast, adoptive transfer of WT cells into *db*/*db* mice (WT→*db*/*db*) resulted in a lower histopathology score. As expected, femora were shorter in *db*/*db* mice than in WT mice (**Panel B**). However, treatment did not normalize femur length. The impact of adoptive transfer of WT bone marrow cells into *db*/*db* mice on distal femur epiphysis can be appreciated in representative micrographs shown in **Panel C**.

## 3. Discussion

Skeletal maturation in the distal femur epiphysis was delayed in *ob*/*ob* and *db*/*db* mice irrespective of sex, food intake (ad libitum or pair-fed to WT), or housing temperature (room temperature or thermoneutral). The osteoclast-rich osteopetrotic skeletal phenotype observed in *ob*/*ob* and *db*/*db* mice was absent in heterozygous *ob*/+ and *db*/+ mice. Interventions known to increase bone resorption in long bones (cold stress, simulated microgravity, focal inflammation) were largely ineffective in advancing skeletal maturation in leptin signaling-deficient mice, whereas leptin replacement was efficacious. Lepr was identified on MCSFR^+^ CD11b^+^ osteoclast precursors in bone marrow and on osteoclasts generated in vitro. Finally, adoptive transfer of Lepr^+^ bone marrow cells into *db*/*db* mice attenuated the osteopetrotic phenotype.

Low leptin levels in individuals with anorexia nervosa are associated with impaired skeletal maturation and growth failure [26,27], reduced peak bone mass, and low bone turnover [28]. Low leptin levels resulting from caloric restriction or due to an inability to generate leptin also impair linear bone growth, maturation, and turnover in rodents [19]. A genetic-based deficiency in leptin synthesis in mice also results in pathological alterations in growth plate architecture, a mild form of osteoclast-rich osteopetrosis, and bone compartment-specific abnormalities in cancellous bone microarchitecture [2,12,29], all correctable by leptin replacement [10,12,20,30,31].

The adipokine leptin is required for efficient replacement of the epiphyseal cartilage template by bone during skeletal maturation [10,12]. However, the mechanisms by which leptin regulates this action remain to be determined. Osteoclast number is either normal or elevated in *ob*/*ob* mice, providing strong evidence that osteoclast activity, essential for removing the calcified cartilage template, is inadequate [10]. Supporting evidence for reduced resorption includes lower biochemical markers of bone resorption in serum and increased retention of fluorochrome labels deposited in bone in leptin-deficient *ob*/*ob* mice [10,20,32]. In the present studies, we identified Lepr on osteoclast precursors in bone marrow and on osteoclasts generated in vitro from bone marrow cells. These results demonstrate the potential for leptin to act directly on osteoclast precursors and/or osteoclasts to regulate osteoclast activity and/or function.

Leptin is a potent positive regulator of linear bone growth in mice [30]. Mechanistically, leptin promotes chondrocyte differentiation and matrix maturation during endochondral ossification [14]. Notably, Kishida et al. [14] reported that the growth plates of *ob*/*ob* mice showed disturbed columnar structure. Although adoptive transfer of Lepr^+^ bone marrow cells into *db*/*db* mice was effective in reducing cartilage within the cancellous compartment of the epiphysis, this treatment did not correct the deficiency in bone length. Hypertrophic chondrocytes in normal and *ob*/*ob* mice express Lepr [14], but our results suggest that adoptive transfer of bone marrow cells does not repopulate growth plate chondrocytes in *db*/*db* mice with Lepr^+^ donor cells. However, this will need to be confirmed in future studies.

The peripheral action of leptin on epiphyseal cartilage was evaluated in the present study following adoptive transfer of Lepr^+^ or Lepr^-^ bone marrow cells into *db*/*db* mice. In contrast to adoptive transfer of Lepr^+^ bone marrow cells into *db*/*db* mice (WT→*db*/*db*), adoptive transfer of Lepr^-^ bone marrow cells into *db*/*db* mice (*db*/*db*→*db*/*db*) had minimal influence on cartilage embedded in trabeculae in the cancellous compartment of the epiphysis, indicating high-dose irradiation, a known stimulator of bone resorption [33], was insufficient to advance skeletal maturity in the distal femur epiphysis. Similarly, strategies known to increase cancellous bone resorption without impacting linear bone growth (simulated microgravity, cold stress, and polyethylene particle-included osteolysis) were likewise ineffective, whereas combination treatment (polyethylene particles plus continuous leptin) and adoptive transfer of Lepr^+^ bone marrow cells into *db*/*db* mice decreased abnormal cartilage in the epiphysis. Taken together, these findings suggest that epiphyseal chondroclasts require leptin to efficiently resorb calcified cartilage. While chondroclasts are morphologically similar to osteoclasts, they can be distinguished from the latter based on discrete transcriptomic features [34]. Functional differences between chondroclasts and osteoclasts may explain why increased resorption of metaphyseal cancellous bone does not advance skeletal maturation in the epiphysis of leptin signaling-deficient mice.

*ob*/*ob* and *db*/*db* mice exhibit many of the defects common to osteopetrosis (stunted linear growth, disruption of growth plate architecture, retention of calcified cartilage, blindness, hearing loss, depressed immune system function, and impaired tooth eruption) [12,35]. A polymorphism in the leptin receptor gene is associated with responsiveness to growth hormone replacement therapy in patients with idiopathic short stature and growth hormone deficiency, suggesting a role for leptin in linear bone growth in humans [36]. However, it remains unclear whether humans with loss-of-function mutations in leptin signaling develop a defect in osteoclast function.

Most defects in osteoclast-rich osteopetrosis are caused by loss-of-function mutations that impair the acidification process through trafficking and/or fusion of lysosome-related organelles to the ruffled border. Mutations in the osteoclast vacuolar proton pump (TCIRG1) and the H+-Cl_2_ exchange transporter 7 (CLCN7) account for the majority of autosomal recessive osteopetrosis, whereas variants encoding the carbonic anhydrase II enzyme (CA2), a stabilizing b-subunit of the H+-Cl_2_ exchange transporter 7 (OSTM), and a lysosome-associated protein involved in vesicular trafficking (PLEKHM1) are less common [35,37]. Furthermore, in addition to biological-based mechanisms, drugs that inhibit bone resorption, such as bisphosphonates, can induce osteoclast-rich osteopetrosis in children [38].

The precise pathways for leptin regulation of osteoclast activity have yet to be established but likely involve coordinated actions of the hormone on multiple genes that in concert regulate osteoclast activity. In support, gene profiling has established that leptin deficiency results in the altered expression of several genes related to chondrocyte and osteoclast differentiation and function [20]. Specifically, leptin deficiency resulted in lower expression levels for *Col10a1*, *Col2a1*, *Csf1*, *Icam1*, *Mmp8*, and *Sp7*. It is notable that the protein products of these genes play essential roles in cartilage remodeling [39]. Biologically based osteopetrosis is classified by mode of inheritance as either autosomal dominant, autosomal recessive, or X-linked recessive. In the present study, we did not detect abnormalities in the epiphysis of *db*/+ mice, suggesting that the osteopetrotic phenotypes of *ob*/*ob* and *db*/*db* mice are expressed in an autosomal recessive manner. This is an important finding because it indicates that the actions of leptin on normal skeletal maturation, although important, are largely permissive.

This study has several limitations. While we provide strong evidence that peripheral leptin signaling is required for normal skeletal maturation in mice and have shown that multiple hematopoietic lineage cells, including osteoclast precursors, B cells, T cells, and osteoclasts, express leptin receptors, we have not established the precise requirements for leptin signaling by these cells in skeletal maturation. Studies performed in *ob*/*ob* and *db*/*db* mice must be interpreted with caution because intrinsic factors such as the propensity for these mice to develop obesity and diabetes, as well as the impact of leptin on thermoregulation and gonadal and immune function could have indirect effects on skeletal maturation. While representative studies were performed in male and female mice, not all assays were performed in both sexes. However, to date, we have not detected sex-specific effects of leptin on bone. To address the generalizability of results, we performed studies using obese diabetic *ob*/*ob* and *db*/*db* mice as well as studies in which we used caloric restriction and thermoneutral housing to prevent excessive weight gain.

In summary, leptin signaling-deficient *db*/*db* mice have delayed epiphyseal maturation due to impaired replacement of the cartilage template by bone, resulting in the retention of chondrocytes as well as cartilage matrix. Osteoclast-lineage cells express the long form of the leptin receptor, Lepr, which we interpret as evidence that leptin has direct actions on these cells. This conclusion is supported by our finding that adoptive transfer of Lepr^+^ bone marrow cells accelerated skeletal maturation in *db*/*db* mice. Taken together, these findings provide supporting evidence that leptin is an important permissive factor in skeletal maturation and that these actions are mediated, at least in part, through hematopoietic lineage cells.

## 4. Methods

The experimental protocols were approved by the Institutional Animal Care and Use Committee at Oregon State University or the University of Florida.

### 4.1. Mice

Mice [C57BL/6J (B6, wildtype, WT), C57BL/6-Tg(CAG-EGFP)1Osb/J, *ob*/*ob*, *ob*/+, *db*/*db*, *db*/*+*] were purchased from Jackson Laboratory (Bar Harbor, ME, USA) and, following a 3- to 7-day acclimation interval, were maintained single-housed at either room temperature (20–23 °C) or thermoneutral temperature (30–32 °C). Water was provided ad libitum to all animals. Mice were fed standard rodent chow (Teklad 8604, Harlan Laboratories, Indianapolis, IN, USA) ad libitum. *ob*/*ob* mice were fed ad libitum or pair-fed to WT mice, depending on the study. Upon completion of the study, mice were anesthetized with isoflurane anesthesia and terminated by decapitation.

### 4.2. Evaluation of Skeletal Maturation of Mouse Long Bones

**Figure 7:**
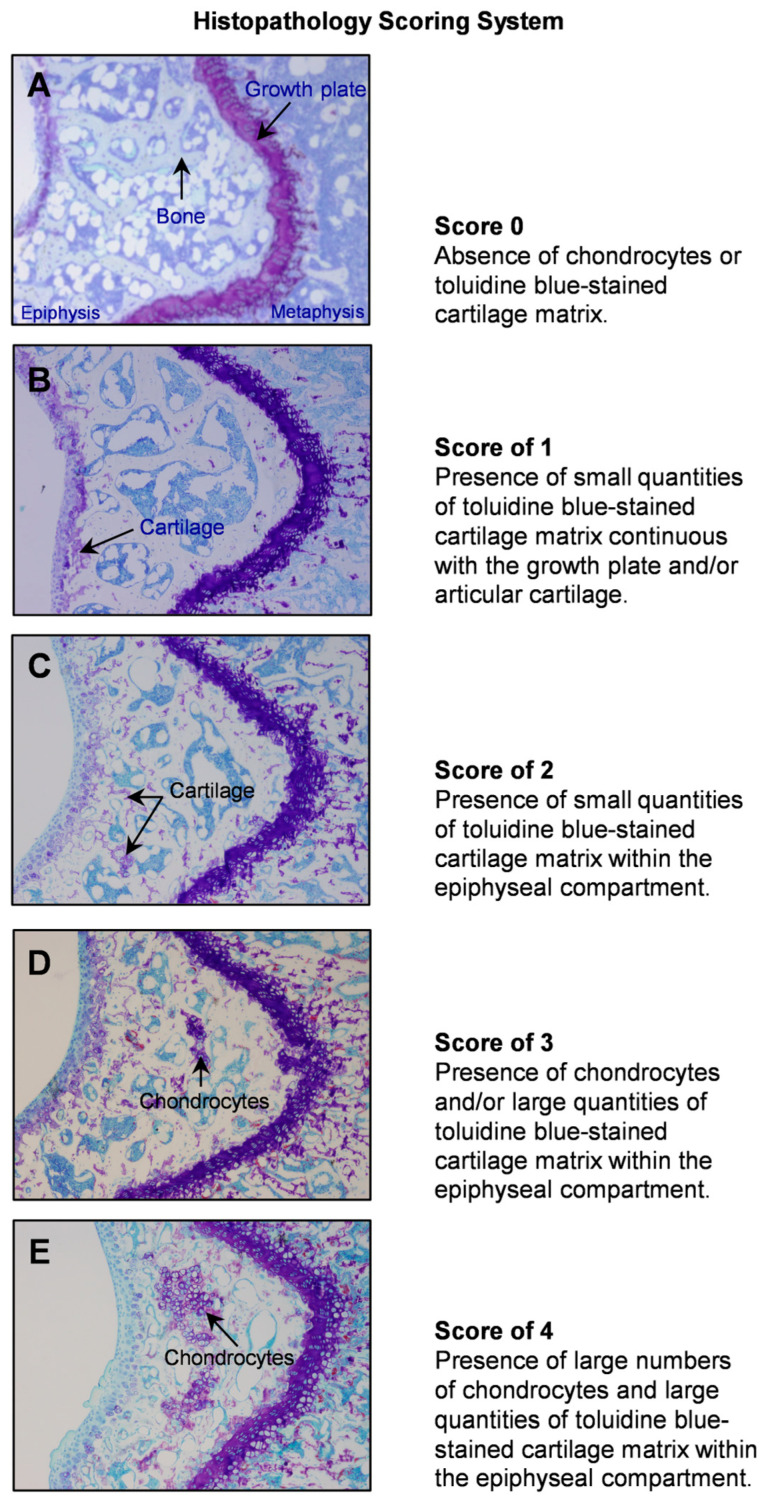
Representative images depicting histopathology score for cartilage retention in the distal femur epiphysis. Scores range from 0 (no cartilage) to 4 (extensive cartilage).

Distal femora were prepared for histomorphometric evaluation as described [40]. In brief, undecalcified distal femora were dehydrated and embedded in modified methyl methacrylate. Frontal sections, 4 µm thick, were cut with a vertical bed microtome (Leica 2065, Wetzlar, Germany) and affixed to gel-coated slides. One slide was stained for tartrate resistant acid phosphatase and counterstained with toluidine blue (pH 2.5) to identify osteoclasts and cartilage matrix, respectively. The cartilage matrix was identified by metachromatic staining of proteoglycans and glycosaminoglycans. The entire cancellous envelope bounded by growth plate and articular cartilage was evaluated in the distal femur epiphysis for skeletal maturation using a histopathology scoring system of epiphyseal cartilage retention. This scoring system (scale of 0 to 4) is illustrated in Figure 7: score of 0: absence of chondrocytes or toluidine blue-stained cartilage matrix (**Figure 7A**); score of 1: presence of small quantities of toluidine blue-stained cartilage matrix continuous with the growth plate and/or articular cartilage (**Figure 7B**); score of 2: presence of small quantities of toluidine blue-stained cartilage matrix within the epiphyseal compartment (**Figure 7C**); score of 3: presence of chondrocytes and/or large quantities of toluidine blue-stained cartilage matrix within the epiphyseal compartment (**Figure 7D**); score of 4: presence of large numbers of chondrocytes and large quantities of toluidine blue-stained cartilage matrix within the epiphyseal compartment (**Figure 7E**). When cartilage content was deemed intermediate between whole number scores, a 0.5 was added to the score. Scoring was performed blinded for all studies by the same observer (RTT). The reproducibility of the scoring system was verified by repeat measurements in a subset of specimens (n = 45): the correlation coefficient was 0.90.

### 4.3. Studies

- Skeletal maturation in the distal femur epiphysis in 16-week-old WT, *ob*/*ob*, *ob*/*+*, *db*/*db*, and *db*/*+* mice.

WT mice approach skeletal maturity by 16 weeks of age [41]. Specifically, at this age there is minimal capacity for further bone elongation and the histopathology score for the distal femur epiphysis is typically <2 (presence of small quantities of toluidine blue-stained cartilage matrix within the epiphyseal compartment). Prior research revealed that *ob*/*ob* and *db*/*db* mice have reduced linear bone growth and delayed skeletal maturation [10,30]. Here, we extend prior studies by (1) confirming that delayed skeletal maturation in the distal femur epiphysis previously reported in male *ob*/*ob* mice also occurs in females (Study A.1), (2) determining the impact of reduced capacity to generate leptin (*ob*/+) [32] on skeletal maturation in the distal femur epiphysis in male mice (Study A.2), and (3) determining the impact of partial leptin receptor deficiency (*db*/+) [42] on skeletal maturation in the distal femur epiphysis in female mice (Study A.3).

• Study A.1. Skeletal maturation in female WT and *ob*/*ob* mice.

Female WT (n = 11) and *ob*/*ob* (n = 10) mice were housed at 32 °C upon arrival at Oregon State University at 5 weeks of age until time of sacrifice at 18 weeks of age. WT mice were fed ad libitum. *ob*/*ob* mice were pair-fed to WT mice (*ob*/*ob* mice were fed an amount of food equivalent to the group mean for WT mice) to minimize differences in weight gain between the two strains [20,23].

• Study A.2. Skeletal maturation in male WT, *ob*/+, and *ob*/*ob* mice.

Male WT (n = 10), *ob*/+ (n = 9), and *ob*/*ob* (n = 9) mice were housed at 32 °C upon arrival at Oregon State University at 4 weeks of age until time of sacrifice at 15 weeks of age. WT and *ob*/+ mice were fed ad libitum. *ob*/*ob* mice were pair-fed to WT mice.

• Study A.3. Skeletal maturation in female WT, *db*/+, and *db*/*db* mice.

Female WT (n = 7), *db*/+ (n = 8), and *db*/*db* (n = 7) mice were housed at 22 °C upon arrival at Oregon State University at 8 weeks of age until time of sacrifice at 17 weeks of age. All mice were fed ad libitum.

B. Skeletal maturation in the distal femur epiphysis in ob/ob mice subjected to interventions that exaggerate bone resorption.

Since leptin-deficient *ob*/*ob* mice have impaired skeletal maturation resulting in mild osteopetrosis, it is possible that interventions that increase bone resorption may attenuate the osteopetrotic phenotype. We therefore evaluated skeletal maturation in *ob*/*ob* mice subjected to cold temperature stress (Study B.1), simulated microgravity; Study B.2), focal inflammation (Study B.3), and focal inflammation plus leptin administration (Study B.4).

• Study B.1. Cold stress-induced bone loss.

A thermoneutral temperature minimizes resting energy expenditure [43,44], increases peak bone mass, and slows cancellous bone loss in male and female mice, in part, by reducing bone resorption [23,45]. We therefore compared the severity of osteopetrosis in the distal femur epiphysis in female *ob*/*ob* mice housed at room temperature (22 °C) to mice housed at a thermoneutral temperature (32 °C). Four groups of mice were studied: WT (n = 9) and *ob*/*ob* (n = 8) mice housed at room temperature (22 °C) and WT (n = 11) and *ob*/*ob* (n = 10) mice housed at a thermoneutral temperature (32 °C). The latter 2 groups of mice were transferred to 32 °C housing at 6 weeks of age, when female B6 mice achieve peak cancellous bone volume fraction in distal femur metaphysis [46]. All animals were fed ad libitum and maintained at their respective housing temperature until 4 months of age.

• Study B.2. Hindlimb unloading-induced bone loss.

Hindlimb unloading (HU), a ground-based model for simulated microgravity, results in rapid bone loss in mouse hindlimbs due to increased bone resorption [47]. In this experiment, male WT and *ob*/*ob* mice were housed at a thermoneutral temperature upon arrival at Oregon State University at 4 weeks of age until sacrifice at 17 weeks of age. At 15 weeks of age, the mice were randomized by body weight into one of four treatment groups (n = 10/group): (1) WT control, (2) WT HU, (3) *ob*/*ob* control, and (4) *ob*/*ob* HU. The mice were HU for 2 weeks and sacrificed. Prior to HU, WT mice were fed ad libitum, and *ob*/*ob* mice were pair-fed to WT mice. During HU, all mice were pair-fed to WT HU mice. The procedure for HU and the effect of HU on bone architecture in these animals have been reported [24].

• Study B.3. Inflammation-induced bone loss.

Polyethylene particles inserted over calvaria induce focal inflammation and calvarial osteolysis. Additionally, focal inflammation induced by polyethylene particles increases bone resorption and induces bone loss in the distal femur [48]. Based on these observations, we evaluated the effect of polyethylene particle-induced calvarial osteolysis on skeletal maturation in the distal femur epiphysis of female *ob*/*ob* mice. Female WT and *ob*/*ob* mice (n = 10/group) were housed at a thermoneutral temperature upon arrival at Oregon State University at 4 weeks of age until sacrifice at 7 weeks of age. At 5 weeks of age, the mice within each genotype were randomized by body weight into one of two groups (n = 5/group): (1) a no-treatment control or (2) particles. The mice were sacrificed 2 weeks following particle implantation (at 7 weeks of age). All mice were fed ad libitum. The procedure for particle insertion and the effect of particle insertion on calvarial osteolysis in these animals have been reported [25].

• Study B.4. Inflammation-induced bone loss and leptin replacement.

Leptin was shown to increase particle-induced osteolysis in *ob*/*ob* mice [25]. The present evaluation was conducted to determine the combined effects of polyethylene particles and leptin treatment on skeletal maturation in the distal femur epiphysis of female *ob*/*ob* mice. WT (n = 19) and *ob*/*ob* (n = 30) mice were housed at a thermoneutral temperature upon arrival at Oregon State University at 4 weeks of age until sacrifice at 8 weeks of age. WT mice were fed ad libitum. From 4 to 6 weeks of age, all *ob*/*ob* mice were pair-fed to WT mice. At 6 weeks of age, the WT mice were randomized by weight into one of two treatment groups: (1) no-treatment control (n = 10) or (2) particles (n = 9), while the *ob*/*ob* mice were randomized into one of four treatment groups: 1) no-treatment control (n = 8), 2) particles (n = 8), (3) particles + continuous leptin (cLeptin, n = 6), or (4) particles + intermittent leptin (iLeptin, n = 8), and particles implanted over calvaria. Following particle implantation, all mice were pair-fed to the WT + particles mice. Mouse leptin (498-OB-05M, R&D Systems, Minneapolis, MN) was delivered continuously (6 µg/d) using subcutaneously implanted osmotic pumps (Alzet Model 1002, Durect Corporation, Cupertino, CA, USA) in the *ob*/*ob* + cLeptin group or intermittently by subcutaneous daily injection (40 µg/d) in the *ob*/*ob* + iLeptin group. The mice were sacrificed at 8 weeks of age, 2 weeks following particle implantation. The effect of particle insertion and leptin treatment on calvarial osteolysis in these animals has been reported [25].

C. Skeletal maturation in the distal femur epiphysis in ob/ob mice following leptin replacement.

Based on the normal epiphyseal cartilage phenotype score in *ob*/+ and *db*/+ mice (Figure 2), the mild osteopetrosis observed with leptin signaling deficiency (*ob*/*ob* and *db*/*db* mice) is likely autosomal recessive. This conclusion is supported by a study demonstrating that long-duration (30 weeks) hypothalamic gene therapy ‘cures’ osteopetrosis in male *ob*/*ob* mice [49]. Also, we showed that bone resorption was increased following treatment with leptin for 2 weeks [11]. To build upon these results, we investigated changes in skeletal maturation in the distal femur epiphysis following short-duration (2 weeks) treatment with subcutaneously administered leptin (Study C.1) and in response to longer duration (15 weeks) hypothalamic leptin gene therapy treatment (Study C.2).

• Study C.1. Short-duration leptin treatment.

Female WT (n = 9) and *ob*/*ob* (n = 47) mice were housed at a thermoneutral temperature upon arrival at Oregon State University at 6 weeks of age until sacrifice at 8 weeks of age. The *ob*/*ob* mice were randomized by weight into one of six treatment groups (n = 7–8 mice/group), with leptin delivered at a dose rate of 0 (vehicle), 4, 12, 40, 140, or 400 ng/h. Vehicle (20 mM Tris-HCL, Invitrogen, Carlsbad, CA, USA) or mouse leptin (498-OB-05M, R&D Systems, Minneapolis, MN) was infused using subcutaneously implanted osmotic pumps (Alzet Model 1002, Durect Corporation, Cupertino, CA, USA) for the 2-week duration of the study. WT mice were untreated. All mice were fed ad libitum. The leptin dose–response effects on bone formation and energy balance in these animals have been reported [11]. Specifically, leptin administration for 2 weeks was shown to reduce food intake and increase bone formation.

• Study C.2. Long-duration hypothalamic leptin gene therapy.

Male WT and *ob*/*ob* mice were housed at room temperature (21–23 °C) under specific pathogen-free conditions upon arrival at the University of Florida at 8–10 weeks of age until time of sacrifice at 23–25 weeks of age. Following 1 week of acclimatization, *ob*/*ob* mice were randomized into one of two treatment groups: (1) recombinant adeno-associated virus-green fluorescent protein (rAAV-GFP, control vector, n = 5) or (2) rAAV-Leptin (n = 9). The rAAV-Leptin and rAAV-GFP vectors were constructed, packaged, and administered as described [50]. WT mice (n = 3) were untreated. All mice were fed ad libitum for the duration of the study. The effect of leptin gene therapy on bone architecture in these animals has been reported [49].

D. Expression of Lepr in hematopoietic lineage cells in WT mice.

Lepr signaling is important in the differentiation and function of a variety of hematopoietic lineage cells [51,52]. While HSCs [53] and immune cells [54], including B and T cells, express Lepr, it is less clear whether osteoclast precursors or osteoclasts express this receptor. We therefore evaluated the expression of Lepr in cell populations derived from the hematopoietic lineage, including B cells, T cells, mast cells, osteoclast precursor cells, and bone marrow-derived osteoclasts. WT mice, 4–8 weeks old, were used as source of spleen and bone marrow cells.

In vitro T cell activation: Single-cell suspension was prepared from spleens, and T cells were cultured in RPMI media containing 10% fetal bovine serum (FBS) and activated with anti-CD3 (5 µg/mL) (ThermoFisher, Waltham, MA, USA) for 48h prior to flow cytometry analysis.

In vitro osteoclast differentiation: Bone marrow cells were cultured in vitro in αMEM media containing 10% FBS, 50 ng/mL M-CSF, and 50 ng/mL RANKL (ThermoFisher, Waltham, MA, USA) for osteoclast differentiation for 7–10 days until large multi-nucleated osteoclasts were observed. Osteoclasts were stained for the presence of tartrate-resistant acid phosphatase (TRAP) using TRAP Staining Kit (Kamiya Biomedical, Seattle, WA, USA). Osteoclast Lepr expression was detected by immunohistochemistry using rabbit anti-Lepr (Abcam, Eugene, OR, USA), followed by AlexaFluor488 goat anti-rabbit IgG (ThermoFisher, Waltham, MA, USA).

In vitro mast cell differentiation and activation: Bone marrow cells were cultured in vitro in RPMI media containing 10% FBS, 3 ng/mL IL-3, and 15 ng/mL stem cell factor (SCF) (ThermoFisher, Waltham, MA, USA) for mast cells differentiation. Cells were passaged for 4 weeks prior to mast cell activation. Mast cells were activated using TLR4 agonist (LPS) or TLR2 agonist (Pam3CSK1) at 0.1 and 1 µg/mL for 24h, followed by flow cytometry.

Flow cytometry: Cells were resuspended in PBS containing 1% FBS and 1 mM EDTA, stained with anti-Lepr antibodies, and gated using antibody markers specific for B cells (CD19), T cells (CD3), osteoclast precursors (MCSFR, CD11b), HSCs (lineage-specific antibody cocktail, Sca1, CD117), and mast cells (FcεRI, CD117). All analyses were performed using an Accuri C6 flow cytometer (BD Bioscience, San Jose, CA, USA).

E. Efficacy of adoptive transfer of bone marrow cells from GFP mice into WT and *db*/*db* mice

Our findings that (1) osteoclast precursors and osteoclasts express Lepr and that (2) skeletal maturation is not impaired in *db*/+ mice suggest it may be feasible to advance skeletal maturation in *db*/*db* mice by adoptively transferring WT bone marrow cells into these animals (WT→db/db). We tested the feasibility of this approach by performing two studies. In the first study (Study E.1), we evaluated the reconstitution of the hematopoietic system following adoptive transfer of bone marrow cells from B6.GFP mice into lethally irradiated WT and *db*/*db* mice. In the second study (Study E.2), we performed in vitro differentiation of osteoclasts and adipocytes from bone marrow, following adoptive transfer. The purpose of this study was to confirm that Lepr^+^ GFP osteoclast precursors differentiate into osteoclasts. Since it is possible that adoptive transfer of unfractionated bone marrow results in mesenchymal as well as hematopoietic stem cell transfer, we also cultured adipocytes (mesenchymal in origin) and assessed for the presence of GFP.

• Study E.1. Reconstitution of hematopoietic compartment following lethal irradiation and bone marrow adoptive transfer.

Four-week-old WT (B6), B6.GFP, and *db*/*db* female mice were used in this study. WT and *db*/*db* mice (n = 3/group) received a lethal irradiation (split dose, 2 × 5 Gy, 3h apart) using a cobalt-60 irradiator source (Radiation Center, Oregon State University). One-day-post-irradiation recipient mice received an adoptive transfer of 2 × 10^6^ B6.GFP bone marrow cells intravenously. The extent of the HSC reconstitution was determined 4 weeks post-adoptive transfer. The percentage of GFP^+^ cells within specific cell populations was determined in bone marrow from femurs (HSCs, osteoclast precursors) and spleen (B cells, T cells) via flow cytometry analysis, as described in the previous section.

• Study E.2. In vitro differentiation of osteoclasts and adipocytes following adoptive transfer of GFP^+^ bone marrow cells.

Four-week-old female WT and 8-week-old female B6.GFP mice were used in this study. WT mice (n = 4) received lethal irradiation (9 Gy) followed by adoptive transfer of 5 × 10^6^ B6.GFP bone marrow cells intravenously. The extent of the reconstitution in the bone marrow precursors for osteoclasts and adipocytes was determined 8 weeks post-adoptive transfer (bone marrow cells from adoptive transfer recipients were used in cell cultures for in vitro osteoclast and adipocyte differentiation). Osteoclast differentiation was performed as described in the previous section. For adipocyte differentiation, bone marrow cells were cultured in αMEM media containing 10% FBS, 10 µM dexamethasone, 5 µM IBMX, and 10ng/mL insulin for 2 weeks. Adipocytes were identified by the presence of oil droplets using Oil Red staining. GFP^+^ cells were detected by immunohistochemistry using rabbit anti-GFP AlexaFluor488 (Molecular Probes, Eugene, OR, USA).

F. Efficacy of adoptive transfer of bone marrow cells from WT and *db*/*db* mice into *db*/*db* mice in advancing skeletal maturation

Following the establishment of the feasibility of restoring Lepr^+^ to hematopoietic cells in *db*/*db* mice, we assessed the impact of adoptive transfer of WT bone marrow cells (Lepr^+^) into *db*/*db* mice (WT→*db*/*db*) on skeletal maturation in the femur epiphysis. Five groups of 8-week-old female mice were evaluated: (1) untreated WT (n = 7), (2) WT + WT donor BM (WT→WT) (n = 7), (3) untreated *db*/*db* (n = 10), (4) *db*/*db* + db/db donor BM (*db*/*db*→*db*/*db*) (n = 4), and (5) *db*/*db* + WT donor BM (WT→*db*/*db*) (n = 4). Transplant recipient mice were lethally irradiated at 9 Gy using a cobalt-60 irradiator source (Radiation Center, Oregon State University) and reconstituted with 1 × 10^7^ donor bone marrow cells as described [10]. The mice were sacrificed at 4 months of age. All mice were housed at room temperature and fed ad libitum for the duration of the study.

### 4.4. Statistics

The randomized studies in the presented experiments were designed to compare two groups (Figure 2), three or more groups (Figure 2, Figure 3, Figure 4 and Figure 7), or as a two-way factorial design (Figure 3). Data were analyzed using one-way or two-way analysis of variance, or with linear models that have separate variance parameters across groups or nonparametric procedures, specifically Wilcoxon–Mann–Whitney, Kruskal–Wallis, and Scheirer–Ray–Hare tests. Model diagnostics included use of the Breusch–Pagan test for homogeneity of variance, plots of residuals versus fitted values, normal quantile plots, and the Anderson–Darling test of normality. The Benjamini and Hochberg method [55] for maintaining the false discovery rate at a maximum of 5% was used to adjust for multiple comparisons. Data analysis was performed using R version 4.12.

## Data Availability

The original contributions presented in this study are included in the article. Further inquiries can be directed to the corresponding author.
